# Genetic diversity in black South Africans from Soweto

**DOI:** 10.1186/1471-2164-14-644

**Published:** 2013-09-23

**Authors:** Andrew May, Scott Hazelhurst, Yali Li, Shane A Norris, Nimmisha Govind, Mohammed Tikly, Claudia Hon, Keith J Johnson, Nicole Hartmann, Frank Staedtler, Michèle Ramsay

**Affiliations:** 1 Division of Human Genetics, School of Pathology, University of the Witwatersrand, Faculty of Health Sciences, Johannesburg, South Africa; 2 Division of Human Genetics, National Health Laboratory Service, Johannesburg, South Africa; 3 Wits Bioinformatics, University of the Witwatersrand, Johannesburg, South Africa; 4 Novartis Institutes for Biomedical Research (NIBR), Human Genetics and Genomics, Cambridge, MA, USA; 5 MRC/Wits Developmental Pathways for Health Research Unit, Department of Paediatrics, School of Clinical Medicine, Faculty of Health Sciences, University of the Witwatersrand, Johannesburg, South Africa; 6 Division of Rheumatology, Chris Hani Baragwanath Hospital and the School of Clinical Medicine, Faculty of Health Sciences, University of the Witwatersrand, Johannesburg, South Africa; 7 Novartis Institutes for Biomedical Research (NIBR), Human Genetics and Genomics, Basel, Switzerland; 8 Sydney Brenner Institute for Molecular Bioscience, University of the Witwatersrand, Johannesburg, South Africa

**Keywords:** African population, Soweto, genetic diversity, southeastern Bantu-speakers, SNPs, SAHGP

## Abstract

**Background:**

Due to the unparalleled genetic diversity of its peoples, Africa is attracting growing research attention. Several African populations have been assessed in global initiatives such as the International HapMap and 1000 Genomes Projects. Notably excluded, however, is the southern Africa region, which is inhabited predominantly by southeastern Bantu-speakers, currently suffering under the dual burden of infectious and non-communicable diseases. Limited reference data for these individuals hampers medical research and prevents thorough understanding of the underlying population substructure. Here, we present the most detailed exploration, to date, of genetic diversity in 94 unrelated southeastern Bantu-speaking South Africans, resident in urban Soweto (Johannesburg).

**Results:**

Participants were typed for ~4.3 million SNPs using the Illumina Omni5 beadchip. PCA and ADMIXTURE plots were used to compare the observed variation with that seen in selected populations worldwide. Results indicated that Sowetans, and other southeastern Bantu-speakers, are a clearly distinct group from other African populations previously investigated, reflecting a unique genetic history with small, but significant contributions from diverse sources. To assess the suitability of our sample as representative of Sowetans, we compared our results to participants in a larger rheumatoid arthritis case–control study. The control group showed good clustering with our sample, but among the cases were individuals who demonstrated notable admixture.

**Conclusions:**

Sowetan population structure appears unique compared to other black Africans, and may have clinical implications. Our data represent a suitable reference set for southeastern Bantu-speakers, on par with a HapMap type reference population, and constitute a prelude to the Southern African Human Genome Programme.

## Background

The African continent continues to attract a growing proportion of research attention due to the unprecedented level of genetic diversity of its peoples [1,2]. In particular, northern and central African countries have been increasingly incorporated into studies assessing human population structure. The Luhya of Kenya, the Maasai of Kinyawa and the Yoruba of Nigeria are well documented in both the HapMap and 1000 Genomes Projects (http://hapmap.ncbi.nlm.nih.gov; http://www.1000genomes.org); the latter of which will also include data pertaining to Gambian (The Gambia), Mende (Sierra Leone) and Esan (Nigeria) populations. The Human Genetic Diversity Project (HGDP) provides genotyping information for populations residing in the Central African Republic, the Democratic Republic of Congo and Senegal [3], whilst independent assessments of Malawian and Ethiopian genetic structure are also available [4,5].

Less well represented in current research, however, are inhabitants of the southern Africa region. Defined here as the collection of Botswana, Lesotho, Swaziland, Namibia and South Africa (according to the United Nations Geoscheme, [6]), southern Africa is home to a predominant population of Bantu-speakers; a sub-group of the Niger-Kordofanian (NK) linguistic group that expanded southwards from Nigeria and Cameroon, beginning approximately five thousand years ago [7,8], reaching South Africa ~1500 to 1000 years ago [9]. Specifically, speakers belong to the “S” group of Bantu language classification [10,11], consisting of mostly Sotho-Tswana, Venda and Nguni languages [12]. The genetic architecture of NK-speakers, in general, has been described as fairly homogeneous [2,13], despite their broad distribution across the continent, however, few studies have sampled extensively from southern African countries. The HGDP includes only a scattering of southern Bantu-speakers from South Africa (eight in total), whilst Tishkoff et al. [2], Xing et al. [14], Schlebusch et al. [15] and Pickrell et al. [16] include limited samples of 41, 27, 20 and 24 such individuals respectively. These individuals were interrogated using a comparatively small selection of genetic markers (with the exception of Schlebusch and colleagues who typed ~2.5 million single nucleotide polymorphisms [SNPs]), restricting the information density. The resulting data are thus not ideal as a suitable reference resource that captures the genetic diversity of the region’s dominant ethnolinguistic group.

The lack of local genetic information with robust allele frequency distributions currently serves as a significant hurdle to designing biomedical research and may have important medical implications. With the highest worldwide prevalence of HIV/AIDS [17] and rising rates of diseases of lifestyle due to rapid urbanisation, southern Africa suffers under the full weight of medical needs, including communicable, non-communicable, perinatal and maternal disorders [18]. According to the World Health Organisation [19], roughly 60% of deaths within southern African countries are attributable to communicable diseases, whilst 30% are caused by non-communicable disorders. Hitherto, investigations into the population-specific genetic causes underpinning these diseases have largely relied on the HapMap reference data for Yoruba and Luhya populations to guide study design. However, the accuracy of this approach remains in doubt, as it is still unclear to what extent tag SNPs from the Yoruba or Luhya can be ported to other Africans [20,21]. Moreover, southern Africans are geographically distant from these proxy populations, resulting in genetic differentiation due to genetic drift, different selection pressures and admixture with different indigenous groups (such as Khoe and San groups) [22]. The generation of local genetic information therefore presents several key benefits in both evaluating the applicability of proxy populations within Africa as well as providing a more accurate reference foundation on which to support future disease research. In addition, it provides a reference from which to identify local founder effects, signatures of selection, levels of admixture and allele frequency variations. Such benefits facilitate the future ideals of personalised medicine, and the knowledge gleaned may well have uses for other populations worldwide, given Africa’s importance for human history. It is these reasons that provided the impetus for the Southern African Human Genome Programme (SAHGP) [23], which ultimately aims to provide a comprehensive, publically available database of genetic information for this region.

As a prelude to the SAHGP, we sought to investigate the genetic diversity amongst urban black South Africans residing in the Soweto-Johannesburg metropolitan area of the Gauteng province - one of the urban centres in South Africa most densely populated by southeastern Bantu-speakers. Soweto is a major contributor to South Africa’s leading rates of urbanization [24], retaining a regular influx of migrant workers (and refugees) since the gold-mining era [25,26], who intermix with local inhabitants. This sets the stage for substantial genetic mixing between separately defined ethnolinguistic subgroups, further complicated by known Caucasian and Indian influences on the area. Accompanying rapid urbanization is a simultaneous transition in epidemiology. For example, the Heart of Soweto study [27] has uncovered distressing statistics that point to a widening spectrum of both traditional forms of infectious heart disease as well as non-communicable forms more commonly seen in developed countries. As a pertinent demonstration, the atherosclerotic disease phenotype that was once largely unobserved amongst black South Africans, was documented in 14% of study cases. Indeed, more than 75% of black South Africans are now considered to possess at least one major risk factor for heart disease [28]. More generally, Mayosi and colleagues [18] reviewed the overall burden of non-communicable disease in South Africa, citing numerous references that demonstrate the increasing prevalence of these diseases. Specifically, they noted the unequal distribution of disease, with the heaviest burden being endured by poor communities in an urban context, as is typical of Soweto. Thus, we aimed to provide a closer examination of genetic variation within Soweto, with the main purpose of providing a more accurate reference dataset for medical and genetic research. We contrasted this variation with selected populations worldwide, with the view of placing southeastern Bantu-speakers in the context of global genetic diversity. Finally, to assess the applicability of such a reference, we sought to determine how similar a larger random sample of black Sowetans was to our own “reference set” by incorporating results from a recent case–control study on rheumatoid arthritis in Soweto, and used the comparison to note certain implications for genomic research in southern Africa.

## Results

### Performance

**Figure 1:**
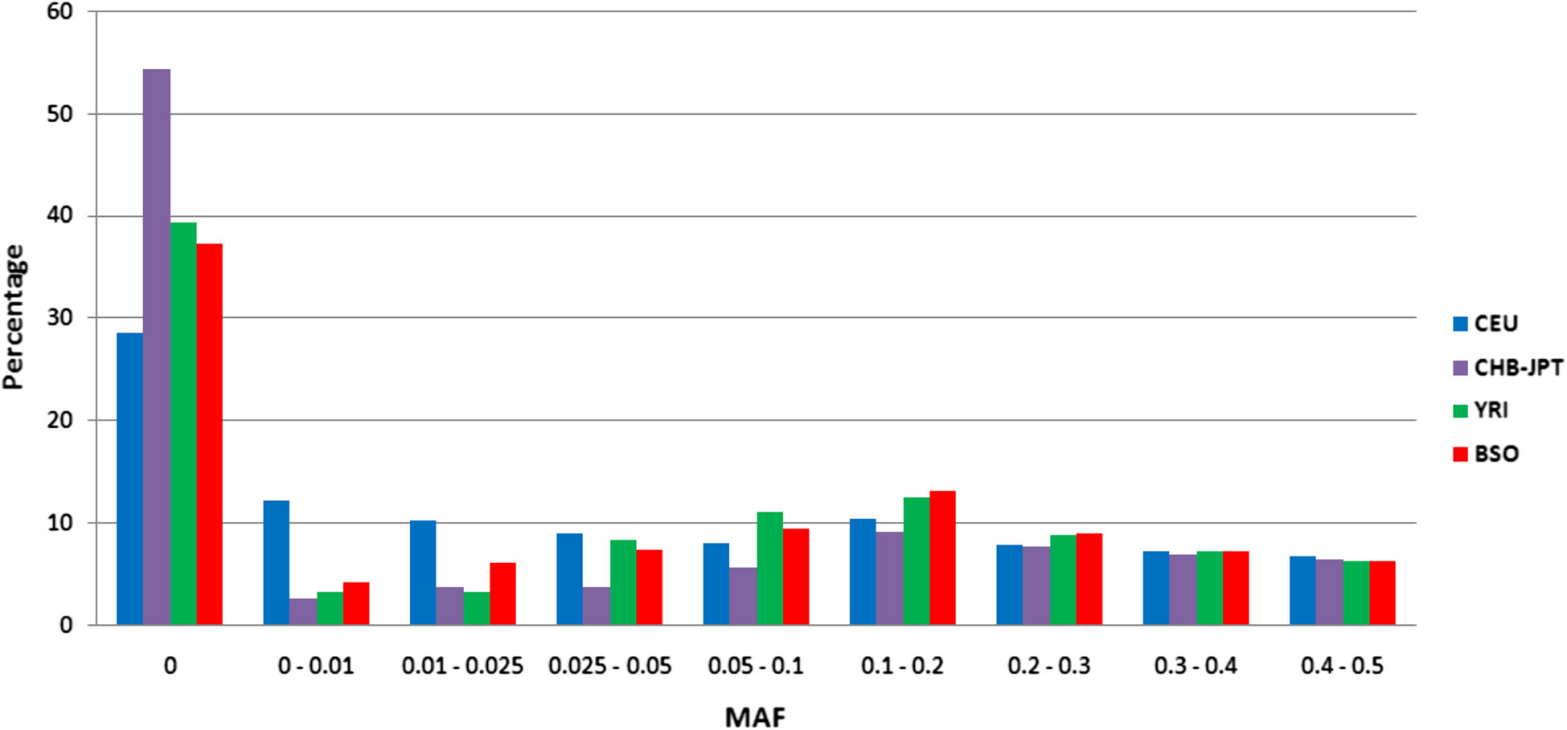
**Minor allele frequency comparison for different populations typed on the Omni5 chip.** We compared the distribution of minor allele frequencies for black Sowetan (BSO; n = 94) individuals to those generated in-house, by Illumina, for the CEU, CHB, JPT and YRI populations. Note that minor allele designation was dependent on genotyping frequencies per population, thus the minor allele per SNP may be different between populations. BSO individuals had an increased fraction of SNPs with minor allele frequencies between 0 and 2.5%, as well as a lower proportion of monomorphic SNPs (0 MAF), when compared to their African counterparts, the Yoruba (n = 55). Between frequencies of 2.5 and 10%, the YRI had a marginally larger fraction of SNPs, but levels remained comparable between the two African groups for common variants with frequencies between 10 and 50%. Performance was best for CEU (n = 113), with a low percentage of monomorphic SNPs and a significantly greater proportion of rare (1-5%) markers. Conversely, Asian [CHB (n = 44) and JPT (n = 40)] populations fared poorly, with over half of all markers on the Omni5 panel lacking variation.

It is commonly accepted that the inadequacy of SNP chips is exposed when used to assess most African populations [29]. Thus, to compare the performance of our samples (BSO – black Sowetans) on the Omni5 chip to other populations, we obtained allele frequency data for the CEU (Utah residents with ancestry from northern and western Europe), YRI (Yoruba in Ibadan, Nigeria), CHB (Han Chinese in Beijing, China) and JPT (Japanese in Tokyo, Japan) HapMap samples that were genotyped on the Omni5 chip, in-house, by Illumina. For each population, the distribution of minor allele frequencies was plotted. Note that minor allele designation was dependent on genotyping frequencies per population, thus the minor allele per SNP may be different between populations. Results are shown in Figure 1. Our samples performed similarly to those of the YRI, with a slighter higher fraction of markers with an allele frequency less than 2.5%, but a lower fraction of markers with a minor allele frequency between 2.5 and 10%, as well as a lower percentage of monomorphic SNPs. A clear bias for low frequency variants was noted for CEU individuals, as SNP selection for the Omni5 was largely based on European data. Asian populations (CHB and JPT) fared least well, with over 50% of markers typed as monomorphic and, therefore, of reduced utility.

### Principal components analysis (PCA)

**Figure 2:**
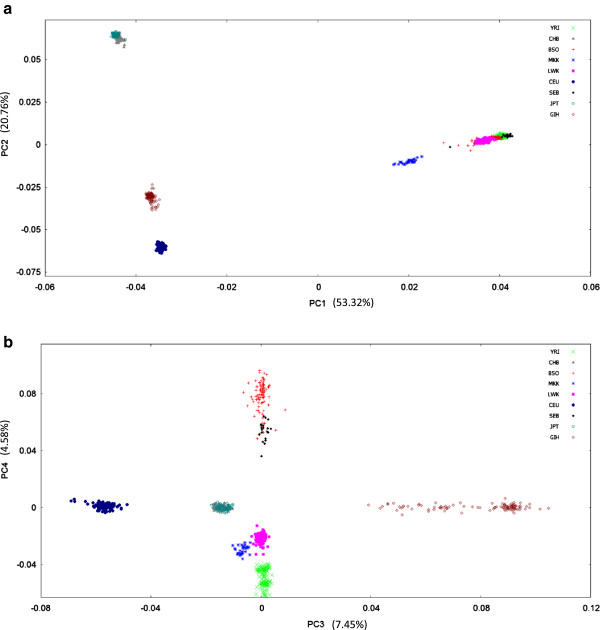
**Intercontinental PCA plots comparing Sowetan genetic variation to populations worldwide.** Sowetan genetic variation was compared to that seen worldwide using principal component analysis. Our data were combined with Omni2.5 data generated as part of the 1000 Genomes Project. We incorporated the main representatives for the European (CEU), Asian (CHB and JPT) and African (LWK, MKK and YRI) continents, as well as Gujarati Indians (GIH) based on reported Indian contributions to the Sowetan gene pool. **a)** Principal components (PC) 1 and 2 divide populations into broad continental clusters, with the exception of GIH. The BSO overlap well with other Africans of the Niger-Kordofanian linguistic group. Nilo-Saharan speaking Maasai are positioned nearby, reflecting the separate history of this linguistic branch. Several BSO individuals separate out from the cluster, indicating possible admixture. **b)** PC3 separates Asian, European and Indian populations, whilst PC4 disaggregates Africans along a north–south gradient. BSO and SEB are clearly distinguished from other black Africans and are more loosely clustered. Plots are based on a panel of 460 568 markers. Refer to Table 1 for sample sizes per population.

**Figure 3:**
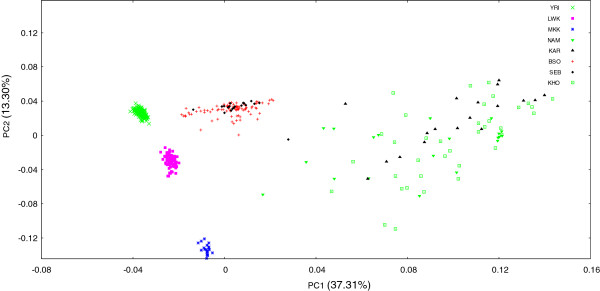
**Intracontinental PCA plot comparing Sowetan genetic variation to other black Africans.** To examine African genetic variation in more detail, a PCA plot was generated that incorporated only BSO, SEB, YRI, MKK, LWK, KAR, KHO and NAM populations. PC1 separated populations along a north–south split, whilst the Nilo-Saharan speaking Maasai separated out along PC2. Khoe-San groups (KAR, KHO and NAM) displayed limited clustering in line with previous reports on their unparalleled genetic diversity. Noticeably, BSO clustering was weaker than that seen in northern Africans, suggesting a greater degree of interindividual variation. Plot was based on a panel of 460 568 markers. Refer to Table 1 for sample sizes per population. BSO - Southeastern Bantu-speakers from the Soweto region; KAR - Karretjie in South Africa; KHO - Khomani in South Africa; LWK - Luhya in Webuye, Kenya; MKK - Maasai in Kinyawa, Kenya; NAM - Nama in Namibia; SEB - Southeastern Bantu-speakers; YRI - Yoruba in Ibadan, Nigeria.

To contextualise Sowetan genetic variation, PCA plots (based on 460 568 SNPs) were generated from the combined dataset (Figures 2 and 3) where data from different population combinations, as well as different principal components are shown. Figures 2a) and 2b) demonstrate intercontinental variation, and include the major African, Asian and European representatives. We included Gujarati Indians in Houston, Texas (GIH) as well, based on historical accounts of Indian influences on the Sowetan gene pool. With respect to principal components (PC) 1 and 2, populations were positioned into broad continental clusters, with the exception of the GIH who clustered separately. BSO individuals clustered along with other black African populations (YRI, LWK and SEB) speaking a Niger-Kordofanian language, whilst the Nilo-Saharan speaking Maasai appear as a distinct cluster. Selected BSO individuals appeared to position spatially in the direction of CEU and GIH populations, reflecting possible admixture.

Principal components 3 and 4 more clearly distinguished African populations from one another. Component 3 highlights the separation between Europeans, Oriental populations and Gujarati Indians, the latter of which appears as an extended cluster. Component 4 disaggregates African populations along a north–south gradient, with a correspondingly clear distinction between Sowetans and the more northern African groups. Southeastern Bantu-speakers (SEB) typed by Schlebusch et al. [15] remained closely paired with BSO, in line with Soweto demographics. In both plots, clustering amongst BSO individuals appeared to be more dispersed compared to other African groups, with a greater overall spread.

To investigate the distinctions between African populations further, we generated an intracontinental plot that included only African populations, namely the BSO, SEB, YRI, MKK, LWK, KAR (Karretjie), KHO (Khomani) and NAM (Nama) (Figure 3). In agreement with typical plots of PC1 versus PC2, black African populations demonstrated a clear separation as a consequence of their geographic distance from each other [14], with PC1 reflecting a north–south split. The Maasai are separated out along PC2, whilst the Khoe-San groups showed limited clustering in accordance with their high genetic diversity [15]. Again, BSO clustering was noticeably weaker than that seen for northern Africans, suggesting a greater degree of interindividual variation.

### Admixture

**Figure 4:**
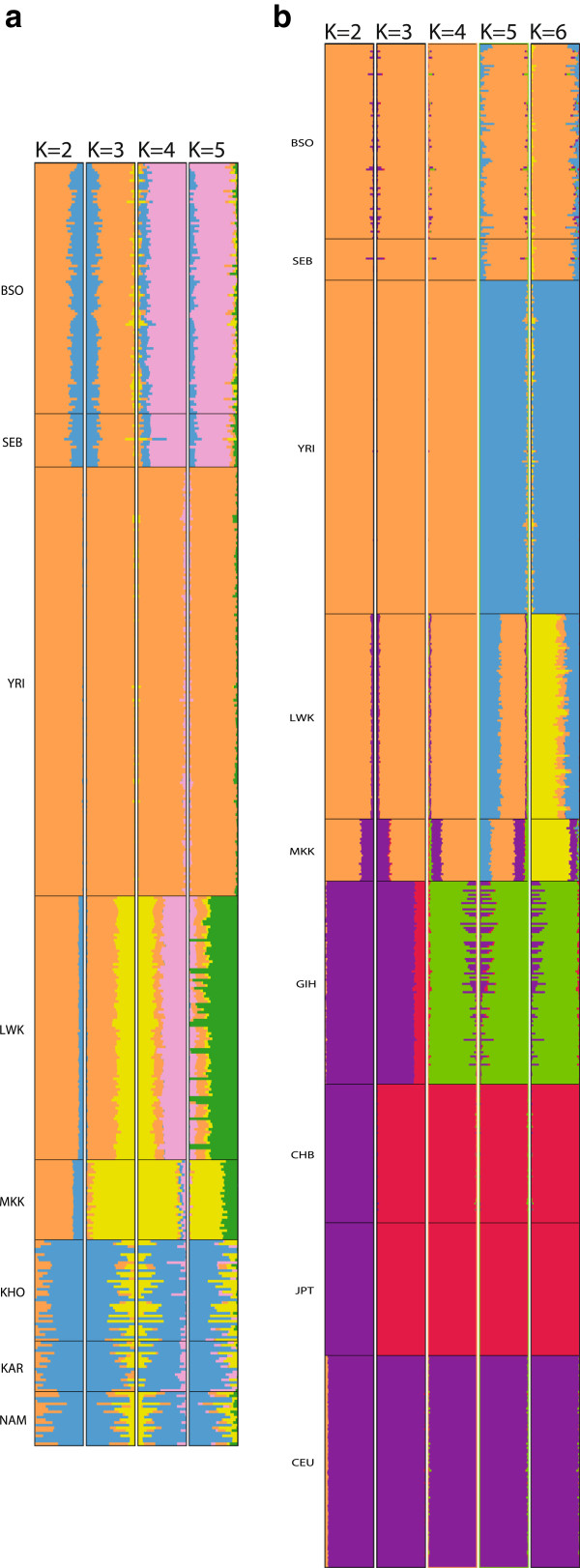
**ADMIXTURE plots comparing genetic variation in Sowetans to that seen worldwide.** ADMIXTURE was used to compare genetic composition of Sowetans to other populations worldwide, based on 460 568 SNP markers. **a)** When incorporating African populations only, the Yoruba (YRI) are distinguished from other Africans from *K*=2. At *K*=3, southeastern Bantu-speakers (BSO and SEB) are discerned from the Luhya (LWK) and Maasai (MKK), but share a degree of ancestry with Khoe-San groups (KAR, KHO, NAM). Both *K*=4 and *K*=5 increasingly depict each African population as a unique entity, in line with the diverse genetic architecture of the continent. **b)** At an intercontinental level, *K*=2 separates Africans from non-Africans whilst *K*=3 groups populations broadly into Asian (CHB, JPT), European (CEU) and African categories. *K*=4 then differentiates Gujarati Indians (GIH) beyond a simple mix of European and Asian genetic variation. Increasing *K* values separate out African populations along the lines described in a). At *K*=6, BSO and SEB appear highly diverse, possessing contributions from all six ancestral clusters.

ADMIXTURE results for ancestral populations *K*=2 to *K*=5 and *K*=2 to *K*=6, for intracontinental and intercontinental datasets respectively, are shown in Figure 4. Intracontinentally (Figure 4a), the present study sample was seen to closely resemble SEB, in confirmation of observed PCA results. From *K*=2, YRI individuals were already distinguished from their African counterparts. By *K*=3, clear separation between BSO, YRI, MKK and LWK populations was evident, along with a clear link between southeastern Bantu-speakers and the southern Khoe-San, in confirmation of previous reports [15,30]. At *K*=5, BSO and SEB presented with greater diversity in admixture than northern Africans. Intercontinentally (Figure 4b), *K*=2 separated Africans from non-Africans, whilst *K*=3 and *K*=4 formed African, European and Asian clusters, with GIH initially shown as having mixed ancestry from both Europe and Asia (*K*=3) before separating as a distinct population at *K*=4. With increasing *K* clusters, African populations are increasingly distinguished. In particular, Bantu-speakers appeared to be significantly different with relatively small contributions from all 6 ancestral populations; a result that was not typical for members of the other populations investigated.

### Sample comparison

**Figure 5:**
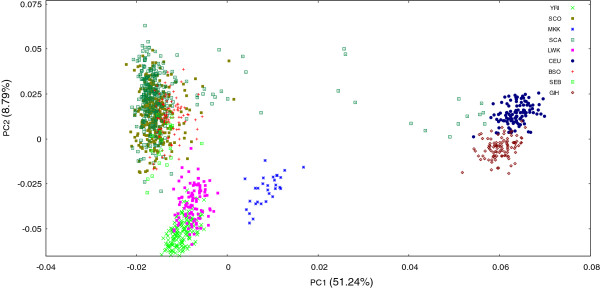
**Principal component analysis of Sowetan cases and controls recruited for a rheumatoid arthritis association study.** To assess the validity of our own sample as a suitable reference for the black Sowetan population, we used PCA to compare results with independently recruited case (SCA; n = 304) and control (SCO; n = 318) samples selected for a rheumatoid arthritis study. Cases and controls matched the clustering pattern of BSO individuals, supporting the use of the latter as a reference sample. However, a minority of cases displayed wider dispersal, with several individuals positioned more closely to Indian and European populations. Plot was based on a panel of 21 412 SNPs. BSO - Southeastern Bantu-speakers from the Soweto region; CEU - Utah residents of European ancestry; GIH - Gujarati Indians from Houston, Texas; LWK - Luhya in Webuye, Kenya; MKK - Maasai in Kinyawa, Kenya; SCA - Black Sowetan case individuals with rheumatoid arthritis; SCO - Black Sowetan control individuals; SEB - Southeastern Bantu-speakers; YRI - Yoruba in Ibadan, Nigeria.

To examine how well our reference Soweto sample represented another larger and independently selected sample of unrelated black Sowetans, we performed a comparison with data from a case–control study for rheumatoid arthritis. PCA results are displayed in Figure 5. We limited our selection of populations outside of the African continent to just CEU and GIH due to reported admixture with BSO. Comparative data were available for 21 412 SNPs. Controls (SCO) from the study closely matched the clustering pattern of the BSO group, suggesting similar overall genetic profiles. Interestingly, the cases (SCA) demonstrated a wider spread of variation, strongly indicative of varied degrees of admixture with Europeans and Indians.

## Discussion

The rapid urbanisation of Soweto and its subsequent epidemiological transition are largely representative of the transformations occurring across the developing southern Africa region [27,31-33]. Consequently, the area’s predominant ethnic group of southeastern Bantu-speakers constitute one of the African continent’s largest health burdens, and understanding their susceptibility to disease, both communicable and non-communicable, grows increasingly important. Progress, however, is hampered by a paucity of genetic data that necessitates the use of proxy populations; an approach with obvious limitations. An appropriate reference dataset would thus greatly improve local research capabilities and obviate the need for proxy genetic data. In the present study, we sought to address the lack of reference data and contrast Sowetan genetic variation to that seen worldwide, and more specifically, within Africa.

Using principal component analysis, we noted two important observations. Firstly, we confirmed that southeastern Bantu-speakers (BSO and SEB) occupy a distinct space from northern Africans. Secondly, we observed a relatively loose clustering of BSO individuals, consistent with the demographic “melting pot” of the urban Soweto community. In confirmation, ADMIXTURE results suggested Sowetans comprise of small contributions from a diverse assortment of ancestral populations, more so than was evident for other African populations investigated, with the exception of the Khoe-San. Such varied contributions, however, were not significant enough to detract from the general homogeneity of the group (consisting of DNA from primarily one ancestral population), suggesting that most migration and admixture into Soweto is likely from areas where individuals have a similar genetic heritage. Consequently, the average level of admixture is unlikely to significantly interfere with the analysis of disease association studies. However, individuals with significant admixture also form part of the Sowetan population [as witnessed in Figure 5]. It is therefore necessary to screen for such individuals and to exclude them from phenotype-genotype association studies, in order to avoid false positive associations as a result of underlying population structure.

Amongst the numerous and diverse sources of genetic variation, Bantu-speakers are specifically known to display levels of Khoe-San admixture [2,34,35]. Our results confirm a degree of admixture between the BSO and the more southerly located Khoe-San (Figure 4a), including the Nama, the Khomani and the Karretjie peoples (whose unsurpassed genetic variation is explored in greater detail elsewhere [15,16]). This admixture likely underpins the weaker clustering of southeastern Bantu-speakers, and uniquely distinguishes them from northern Africans. Indeed, the separation observed between NK-speaking populations included in the present study highlights some of the key benefits to improved marker density and more focused comparisons between populations when assessing genetic structure. Although fairly homogenous when considered on a global scale [2], our comparisons at the intracontinental level revealed significant heterogeneity between western (YRI), central (LWK) and southern (BSO) NK-speakers. Both PCA and ADMIXTURE analyses suggest BSO are dissimilar from the populations commonly used as their proxy (YRI and LWK), with greater interindividual genetic variation. These findings support the use of more detailed assessments of population genetic structure to improve the resolution between closely related, but nonetheless distinct groups of individuals. Moreover, they augment the value of local genetic information, especially when researching the more innately diverse African populations.

In confirmation that our sample was a good representation of the larger Soweto population, we investigated its similarity to a sample of over 600 individuals from a recent rheumatoid arthritis case–control study (Govind et al. in preparation). The cases and controls were separately identified and in the comparisons, the controls clustered tightly with the BSO group, reflecting their common origin, and thus strengthening the applicability of our data as a suitable reference for Soweto and South Africa [according to Statistics South Africa [36], the 2011 Census demonstrated that Gauteng closely mirrors the relative distribution of speakers of the nine Bantu-languages in South Africa as a whole]. Interestingly, among the cases, the majority clustered closely with the BSO, but a minority displayed significant admixture, with a wider spread of genetic variation, despite being selected on the grounds of self-reported black ethnicity. To what reason this wider dispersal is owed remains unclear. Most likely, the more admixed individuals within the group are not permanent residents of the Soweto region, but may have been referred from other locations in order to receive specialised medical treatment beyond the scope of local clinics. Controls were all workers at the hospital (cleaners, nurses, clerks etc.), and thus more inclined to reside permanently in Soweto. The more divergently clustering individuals with significant Indian and Caucasian admixture were removed from the rheumatoid arthritis association study before analysis (Govind et al. in preparation), according to quality control procedures. However, information on divergent and significant admixture in specific individuals is not typically available to health care professionals, and may have important health-related implications since self-reported ethnicity may be used to guide medical advice, including the prescription of drugs. Numerous studies have already reported on certain locus specific population effects concerning drug metabolism, particularly for drugs used to treat cancer and HIV [37-40]. This comparison thus emphasises the value of obtaining local genetic information to highlight ethnic nuances of potentially important clinical relevance.

The performance of the Omni5 in assessing African genetic variation merits comment. Based on our comparisons, the platform performs well in typing common variation in Africans, and will have use in genome-wide association studies. Beneficially, the superior marker density improves the chances for positive associations, which are more likely to progress to the identification of causal variants due to the limited linkage disequilibrium (LD) of African populations [20]. Conversely, limited LD may result in poor detection of association, compounded by the lack of private African alleles on the platform. Regardless, true progress in meeting the medical demands of southeastern Bantu-speakers, and indeed all Africans, will be subordinate to an increased collection of complete genome sequences, which will further outline unique African variation and facilitate the improved stratification of individuals by genetic composition. For example, targeted resequencing of the *CYP3A4* gene in a sample comprised of Khoe-San, Xhosa and Mixed Ancestry individuals from South Africa identified 24 SNPs, two of which were novel, non-synonymous variants [41]. Only one third (8/24) of these variants are included on the Omni5 chip, whilst the novel variation is likely to be private to the African continent, suggesting that full genome sequencing of black Africans will be a necessity if we are to enhance our understanding of the genetic architecture of these peoples. Beyond population stratification, a more thorough appreciation of confounding environmental factors will also need to be fostered [42], especially given the spectrum of living conditions on the continent; from arid to tropical and from rural to urban [1]. Despite these concerns, as one of the most comprehensive genotyping chips currently available, the Omni5 represents a good option for those wishing to pursue GWAS in African populations, based on the performance levels we have witnessed here.

Several limitations to the present study are acknowledged. Ideally, a larger sample size and complete genome sequences would more accurately reflect the full spectrum of genetic diversity across southeastern Bantu-speakers. Our sample of 94 individuals does, however, compare in size to those of the HapMap and 1000 Genomes Projects, which have more than demonstrated their value as reference panels for specific populations. The comparison to Sowetans in the rheumatoid arthritis study was done primarily with markers related to loci relevant to autoimmune disease, which may have introduced some bias, since they may have been involved in significant selection pressures as highlighted by Schlebusch et al. [15]. Lastly, the Illumina Omni5 chip is subject to an ascertainment bias for SNP selection, favouring those polymorphic in European populations, and thus potentially distorting some of the conclusions drawn [43]. In addition, the Omni5 was designed to assess mostly common variants in European populations with a frequency greater than 1%, meaning that the characterisation and distribution of rare variants is still to be incorporated in the assessment of Sowetan genetic structure.

Our data have begun to address the paucity of southern Africa genetic information, although considerable work remains in sampling more broadly across the region. Numerous other ethnicities, including the Cape Mixed Ancestry, southwestern Bantu-speakers (Herero) and Afrikaner populations *inter alia*, present interesting genetic diversity in their own rights, distinct from that seen amongst the more populous southeastern Bantu-speakers. Several studies have already commenced with the documentation of this variation [15,30,44], but it is the larger aim of the SAHGP to provide more thorough reference databases on par with those available for selected populations participating in the International HapMap and 1000 Genomes Projects. Forthwith, the data of the present study may, therefore, be considered the southeastern Bantu-speaker equivalent of a HapMap reference for this population. Future studies will aim to mine these data further, attempting to extract information of particular biomedical relevance.

## Conclusions

To conclude, our investigative search into Sowetan genetic variation is, to date, the most detailed of its kind. We have observed a distinct genetic profile for these individuals, different from other more widely studied African populations, supported by principal component analysis as well as ADMIXTURE. Combined, these results aligned well with demographic and historical knowledge on the inhabitants of the Soweto region, clearly highlighting the significant, but relatively small genetic contributions from far and wide, that have been made to the local gene pool. We have demonstrated that this dataset is a good reference sample for future research on black South Africans who speak southeastern Bantu languages. Most importantly, some of the implications for future medical policy and research are highlighted. Lastly, the dataset may be considered a first step toward the SAHGP, and is available at http://sbimb.core.wits.ac.za/data/SNPgenotyping_01.html.

## Methods

### Samples

Study participants included 94 unrelated southeastern Bantu-speaking South African individuals (43 males and 51 females), residing in the Soweto-Johannesburg metropolitan area whose ethnicity was captured from municipal birth notification forms. These individuals are existing participants in a longitudinal birth cohort and were all born in 1990 [45]. Following informed consent, a 10ml sample of venous blood was drawn, and DNA was extracted using the salting-out procedure [46]. Extracted DNA was normalized to 50ng/μl, in TE buffer. This study was approved by the University of Witwatersrand, Human Research Ethics Committee (Medical) – clearance number M110744.

### Genotyping

Participants were genotyped using Infinium Omni5 beadchips (Illumina, San Diego, USA). DNA samples were prepared in accordance with the Infinium LCG assay (Part # 15025908, Revision A, June 2011 – available from http://www.illumina.com/support/documentation.ilmn). Beadchips were scanned on the Illumina iScan (Illumina, San Diego, USA). Raw data were inspected using Genomestudio (version 2011.1) and genotype calls were made based on a clustering manifest supplied by Illumina.

### Quality control

PLINK [47] was used to assess genotyping quality according to the protocol published by Anderson and colleagues [48]. Samples were checked for discordant sex information (mismatches between documented sex and that suggested by genotyping data), outlying heterozygosity (more than 3 standard deviations from the mean), elevated rates of missing data (genotyping failure rate > 3%), and possible relatedness (identity by descent score > 0.185). Individual SNPs (4 240 992 in total) were checked for excess missingness (missing call-rate above 3%), and markers with a minor allele frequency less than 1% (including monomorphic SNPs) and/or a Hardy-Weinberg equilibrium *P*-value less than 1 × 10^-4^ were removed. Additionally, all X, Y and mitochondrial SNPs were removed, along with those with unknown chromosome location, leaving 2 417 298 markers prior to merging.

### Public datasets

For comparative purposes, we obtained publicly available Omni2.5 chip data from the 1000 Genomes Project (1kGP) (2012/01/31 release). We also obtained genotyping data for southeastern Bantu-speakers and the southern Khoe-San groups from Schlebusch and colleagues (2012) (see Table 1). We limited our selection of Khoe-San groups to those more southerly located as they appear to share more admixture with southeastern Bantu-speakers. Southwestern Bantu-speakers (Herero) were excluded due to a limited sample size (8). These datasets were individually assessed by the same quality control protocol listed above, resulting in 1 500 508 and 1 773 030 high-quality markers for the 1kGP and Schlebusch et al. datasets respectively. These data were then merged to the present study data using PLINK. SNPs that were mismatched for strand were flipped where possible and A/T and C/G markers were removed. After merging, markers with a genotyping success rate lower than 95% were removed to ensure that only overlapping markers between datasets were retained. The final SNP panel consisted of 460 568 markers.

**Table 1:** **Population data**^**1 **^**used for the present study**

**Source**	**Population**	**Description**	**n**^**2**^	**Reference**
Present study	BSO	Southeastern Bantu-speakers from the Soweto region	94	
1000 Genomes Project	CEU	Utah residents of European ancestry	102	1000 Genomes Project (http://ftp.1000genomes.ebi.ac.uk/vol1/ftp/technical/working/20120131_omni_genotypes_and_intensities)
	CHB	Han Chinese in Beijing, China	67	
	JPT	Japanese in Tokyo, Japan	64	
	GIH	Gujarati Indians from Houston, Texas	98	
	YRI	Yoruba in Ibadan, Nigeria	161	
	LWK	Luhya in Webuye, Kenya	99	
	MKK	Maasai in Kinyawa, Kenya	30	
Schlebusch and colleagues	KAR	Karretjie in South Africa	19	Schlebusch et al. 2012
	KHO	Khomani in South Africa	38	
	NAM	Nama in Namibia	20	
	SEB	Southeastern Bantu-speakers	20	

^1^With the exception of the present study, all data were generated through the Illumina Omni 2.5 platform. ^2^Number of individuals in the test sample.

A subset of our results was also compared to those from a recent study on rheumatoid arthritis (Govind et al. in preparation). Briefly, 304 affected individuals and 318 healthy controls (all sourced from a Sowetan-based hospital) were typed on the Illumina Infinium Immunochip (Illumina, San Diego, USA) [49], consisting of ~196 000 genetic variants known to pertain to autoimmune disorder susceptibility. As before, genotyping success thresholds were imposed in order to retain only overlapping markers between the Omni5 and Immunochips, resulting in a final panel of 21 412 SNP markers.

### Data analysis

PLINK was used to generate the necessary minor allele frequency statistics that allowed the assessment of the performance of BSO samples on the Omni5 chip. To compare variation between populations, the *smartpca.perl* script, part of the EIGENSTRAT suite (version 3.0; Helix Systems, Maryland, USA), was used to calculate Eigen vectors that determined the relative principal components. These components were then plotted using Gnuplot (version 4.6) [50]. ADMIXTURE (version 1.22) [51], CLUMPP (version 1.1.2) [52] and Distruct (version 1.1) [53] were used in combination to produce plots for *K*=2 to *K*=6 ancestral populations where applicable, calculated from 100 permutations. To ensure no bias was introduced into the PCA analysis due to variations in sample size, we conducted 50 random samplings of 50 individuals from each population studied (the Khoe-San were treated as a single group). Inter- and intracontinental PCAs using these subsamples demonstrated negligible variation in general patterning and clustering when compared to PC analysis of the full sample sizes (data not shown).

## Abbreviations

1kGP: 1000 Genomes project; BSO: Black southeastern Bantu-speakers in Soweto, Johannesburg; CEU: Utah residents with ancestry from northern and western Europe; CHB: Han Chinese in Beijing, China; GIH: Gujarati Indians from Houston, Texas; GWAS: Genome-wide association study; JPT: Japanese in Tokyo, Japan; KAR: Karretjie in South Africa; KHO: Khomani in South Africa; LD: Linkage disequilibrium; LWK: Luhya in Webuye, Kenya; MKK: Maasai in Kinyawa, Kenya; NAM: Nama in Namibia; NK: Niger-Kordofanian; PC: Principal component; PCA: Principal component analysis; SCA: Black Sowetan case individuals with rheumatoid arthritis; SCO: Black Sowetan control individuals; SEB: Southeastern Bantu-speakers; SNP: Single nucleotide polymorphism; YRI: Yoruba in Ibadan, Nigeria.

## Competing interests

The authors declare that they have no competing interests.

## Authors’ contributions

AM assisted with the genotyping and quality control, performed the data analysis and drafted the manuscript. SH contributed in a major way to the quality control of the data and public datasets, as well as the data analysis with regard to PCA and ADMIXTURE. YL and CH oversaw and implemented early quality control procedures on the raw Omni5 data. NG and MT provided the rheumatoid arthritis data. SAN contributed the BSO samples and KJJ, NH, FS and SAN, provided expertise on the experimental platform and critical comment on the manuscript. MR conceived the study design and was involved in guiding the analysis and critical revisions of the drafted manuscript. All authors read and approved the final manuscript.

## References

[B1] Ramsay M Africa: continent of genome contrasts with implications for biomedical research and health FEBS Lett 2012 586 2813 2819 10.1016/j.febslet.2012.07.061 22858376

[B2] Tishkoff SA Reed FA Friedlaender FR Ehret C Ranciaro A Froment A Hirbo JB Awomoyi AA Bodo JM Doumbo O The genetic structure and history of Africans and African Americans Science 2009 324 1035 1044 10.1126/science.1172257 19407144 PMC2947357

[B3] Cann HM de-Toma C Cazes L Legrand MF Morel V Piouffre L Bodmer J Bodmer WF Bonne-Tamir B Cambon-Thomsen A A human genome diversity cell line panel Science 2002 296 261 262 11954565 10.1126/science.296.5566.261b

[B4] Joubert BR North KE Wang Y Mwapasa V Franceschini N Meshnick SR Lange EM Comparison of genome-wide variation between Malawians and African ancestry HapMap populations J Hum Genet 2010 55 366 374 10.1038/jhg.2010.41 20485449 PMC2909738

[B5] Pagani L Kivisild T Tarekegn A Ekong R Plaster C Gallego Romero I Ayub Q Mehdi SQ Thomas MG Luiselli D Ethiopian genetic diversity reveals linguistic stratification and complex influences on the Ethiopian gene pool Am J Hum Genet 2012 91 83 96 10.1016/j.ajhg.2012.05.015 22726845 PMC3397267

[B6] United Nations Statistics Division - Standard Country and Area Codes Classifications (M49) http://unstats.un.org/unsd/methods/m49/m49regin.htm

[B7] Blench R Archaeology, Language and the African Past 2006 New York: Rowman & Littlefield Publishers Inc.

[B8] Campbell MC Tishkoff SA The evolution of human genetic and phenotypic variation in Africa Curr Biol 2010 20 R166 173 10.1016/j.cub.2009.11.050 20178763 PMC2945812

[B9] Ehret C An African Classical Age: Eastern and Southern Africa in World History, 1000 B.C. to A.D. 400 1998 USA: The University Press of Virginia

[B10] Guthrie M The classification of the Bantu languages 1948 London: Oxford University Press for the International African Institute

[B11] NUGL Online: The online version of the New Updated Guthrie List, a referential classification of the Bantu languageshttp://goto.glocalnet.net/mahopapers/nuglonline.pdf

[B12] Lane AB Soodyall H Arndt S Ratshikhopha ME Jonker E Freeman C Young L Morar B Toffie L Genetic substructure in South African Bantu-speakers: evidence from autosomal DNA and Y-chromosome studies Am J Phys Anthropol 2002 119 175 185 10.1002/ajpa.10097 12237937

[B13] Veeramah KR Wegmann D Woerner A Mendez FL Watkins JC Destro-Bisol G Soodyall H Louie L Hammer MF An early divergence of KhoeSan ancestors from those of other modern humans is supported by an ABC-based analysis of autosomal resequencing data Mol Biol Evol 2012 29 617 630 10.1093/molbev/msr212 21890477 PMC3258037

[B14] Xing J Watkins WS Shlien A Walker E Huff CD Witherspoon DJ Zhang Y Simonson TS Weiss RB Schiffman JD Toward a more uniform sampling of human genetic diversity: a survey of worldwide populations by high-density genotyping Genomics 2010 96 199 210 10.1016/j.ygeno.2010.07.004 20643205 PMC2945611

[B15] Schlebusch CM Skoglund P Sjodin P Gattepaille LM Hernandez D Jay F Li S De-Jongh M Singleton A Blum MG Genomic variation in seven Khoe-San groups reveals adaptation and complex African history Science 2012 338 374 379 10.1126/science.1227721 22997136 PMC8978294

[B16] Pickrell JK Patterson N Barbieri C Berthold F Gerlach L Guldemann T Kure B Mpoloka SW Nakagawa H Naumann C The genetic prehistory of southern Africa Nat Commun 2012 3 1143 23072811 10.1038/ncomms2140 PMC3493647

[B17] Shisana O Rehle T Simbayi LC Zuma K Jooste S Pillay-van-Wyk V Mbelle N Van-Zyl J Parker W Zungu NP South African national HIV prevalence, incidence, behaviour and communication survey 2008: A turning tide among teenagers? 2009 Cape Town: HSRC Press

[B18] Mayosi BM Flisher AJ Lalloo UG Sitas F Tollman SM Bradshaw D The burden of non-communicable diseases in South Africa Lancet 2009 374 934 947 10.1016/S0140-6736(09)61087-4 19709736

[B19] NCD Country Profileshttp://www.who.int/gho/countries/en/

[B20] Conrad DF Jakobsson M Coop G Wen X Wall JD Rosenberg NA Pritchard JK A worldwide survey of haplotype variation and linkage disequilibrium in the human genome Nat Genet 2006 38 1251 1260 10.1038/ng1911 17057719

[B21] Teo YY Small KS Kwiatkowski DP Methodological challenges of genome-wide association analysis in Africa Nat Rev Genet 2010 11 149 160 20084087 10.1038/nrg2731 PMC3769612

[B22] Handley LJ Manica A Goudet J Balloux F Going the distance: human population genetics in a clinal world Trends Genet 2007 23 432 439 10.1016/j.tig.2007.07.002 17655965

[B23] Pepper MS Launch of the Southern African Human Genome Programme S Afr Med J 2011 101 287 288 21837864 10.7196/samj.4860

[B24] Richter LM Panday S Norris SA Factors influencing enrollment: a case study from Birth to Twenty, the 1990 birth cohort in Soweto-Johannesburg Eval Program Plann 2009 32 197 203 10.1016/j.evalprogplan.2008.12.002 19167071 PMC2708337

[B25] Pirie GH da-Silva M Hostels for African migrants in greater Johannesburg Geo Journal 1986 12 173 180

[B26] Wentzel M Tlabela K Kok PC, Gelderblom D, Oucho JO, Van-Zyl J Historical background to South African migration Migration in South and Southern Africa: Dynamics and Determinants 2006 Cape Town, South Africa: HSRC Press 71 96

[B27] Sliwa K Wilkinson D Hansen C Ntyintyane L Tibazarwa K Becker A Stewart S Spectrum of heart disease and risk factors in a black urban population in South Africa (the Heart of Soweto Study): a cohort study Lancet 2008 371 915 922 10.1016/S0140-6736(08)60417-1 18342686

[B28] Tibazarwa K Ntyintyane L Sliwa K Gerntholtz T Carrington M Wilkinson D Stewart S A time bomb of cardiovascular risk factors in South Africa: results from the Heart of Soweto Study “Heart Awareness Days” Int J Cardiol 2009 132 233 239 10.1016/j.ijcard.2007.11.067 18237791

[B29] Schuster SC Miller W Ratan A Tomsho LP Giardine B Kasson LR Harris RS Petersen DC Zhao F Qi J Complete Khoisan and Bantu genomes from southern Africa Nature 2010 463 943 947 10.1038/nature08795 20164927 PMC3890430

[B30] de-Wit E Delport W Rugamika CE Meintjes A Moller M van-Helden PD Seoighe C Hoal EG Genome-wide analysis of the structure of the South African Coloured Population in the Western Cape Hum Genet 2010 128 145 153 10.1007/s00439-010-0836-1 20490549

[B31] Crush J Frayne B McLachlan M Rapid Urbanization and the Nutrition Transition in Southern Africa 2011 Kingston and Cape Town: Queen’s University and AFSUN

[B32] Hay SI Guerra CA Tatem AJ Atkinson PM Snow RW Tropical infectious diseases: Urbanization, malaria transmission and disease burden in Africa Nat Rev Microbiol 2005 3 81 90 10.1038/nrmicro1069 15608702 PMC3130901

[B33] Kruger R Kruger HS Macintyre UE The determinants of overweight and obesity among 10- to 15-year-old schoolchildren in the North West Province, South Africa - the THUSA BANA (Transition and Health during Urbanisation of South Africans; BANA, children) study Public Health Nutr 2006 9 351 358 10.1079/PHN2006849 16684387

[B34] Nurse GT Weiner JS Jenkins T The peoples of Southern Africa and their affinities 1985 Oxford: Clarendon Press

[B35] Thorp CR Hunter-Gatherers and farmers: an enduring frontier in the Caledon Valley 2000 South Africa: Publishers of British Archaeological Reports

[B36] Interactive data (Census, 2011) - SuperWEBhttp://interactive.statssa.gov.za/superweb/login.do

[B37] Dandara C Lombard Z Du-Plooy I McLellan T Norris SA Ramsay M Genetic variants in CYP (-1A2, -2C9, -2C19, -3A4 and -3A5), VKORC1 and ABCB1 genes in a black South African population: a window into diversity Pharmacogenomics 2011 12 1663 1670 10.2217/pgs.11.106 22118051

[B38] Swart M Ren Y Smith P Dandara C ABCB1 4036A>G and 1236C>T Polymorphisms Affect Plasma Efavirenz Levels in South African HIV/AIDS Patients Front Genet 2012 3 236 23133441 10.3389/fgene.2012.00236 PMC3488761

[B39] Swart M Skelton M Wonkam A Kannemeyer L Chin’ombe N Dandara C CYP1A2, CYP2A6, CYP2B6, CYP3A4 and CYP3A5 Polymorphisms in Two Bantu-Speaking Populations from Cameroon and South Africa: Implications for Global Pharmacogenetics Curr Pharmacogenet Personalized Medicineomic 2012 10 43 53 10.2174/1875692111201010043

[B40] van-Schaik RH CYP450 pharmacogenetics for personalizing cancer therapy Drug Resist Updat 2008 11 77 98 10.1016/j.drup.2008.03.002 18486526

[B41] Drogemoller B Plummer M Korkie L Agenbag G Dunaiski A Niehaus D Koen L Gebhardt S Schneider N Olckers A Characterization of the genetic variation present in CYP3A4 in three South African populations Front Genet 2013 4 17 23423246 10.3389/fgene.2013.00017 PMC3574981

[B42] Vilhjalmsson BJ Nordborg M The nature of confounding in genome-wide association studies Nat Rev Genet 2013 14 1 2 23165185 10.1038/nrg3382

[B43] Albrechtsen A Nielsen FC Nielsen R Ascertainment biases in SNP chips affect measures of population divergence Mol Biol Evol 2010 27 2534 2547 10.1093/molbev/msq148 20558595 PMC3107607

[B44] Greeff JM Deconstructing Jaco: genetic heritage of an Afrikaner Ann Hum Genet 2007 71 674 688 10.1111/j.1469-1809.2007.00363.x 17521310

[B45] Richter L Norris S Pettifor J Yach D Cameron N Cohort Profile: Mandela’s children: the 1990 Birth to Twenty study in South Africa Int J Epidemiol 2007 36 504 511 10.1093/ije/dym016 17355979 PMC2702039

[B46] Miller SA Dykes DD Polesky HF A simple salting out procedure for extracting DNA from human nucleated cells Nucleic Acids Res 1988 16 1215 10.1093/nar/16.3.1215 3344216 PMC334765

[B47] Purcell S Neale B Todd-Brown K Thomas L Ferreira MA Bender D Maller J Sklar P de-Bakker PI Daly MJ Sham PC PLINK: a tool set for whole-genome association and population-based linkage analyses Am J Hum Genet 2007 81 559 575 10.1086/519795 17701901 PMC1950838

[B48] Anderson CA Pettersson FH Clarke GM Cardon LR Morris AP Zondervan KT Data quality control in genetic case–control association studies Nat Protoc 2010 5 1564 1573 10.1038/nprot.2010.116 21085122 PMC3025522

[B49] Cortes A Brown MA Promise and pitfalls of the Immunochip Arthritis Res Ther 2011 13 101 10.1186/ar3382 21345260 PMC3157635

[B50] Williams T Kelley C Gnuplot 4.6: An Interactive Plotting Program 2004

[B51] Alexander DH Novembre J Lange K Fast model-based estimation of ancestry in unrelated individuals Genome Res 2009 19 1655 1664 10.1101/gr.094052.109 19648217 PMC2752134

[B52] Jakobsson M Rosenberg NA CLUMPP: a cluster matching and permutation program for dealing with label switching and multimodality in analysis of population structure Bioinformatics 2007 23 1801 1806 10.1093/bioinformatics/btm233 17485429

[B53] Rosenberg NA DISTRUCT: a program for the graphical display of population structure Mol Ecol Notes 2004 4 137 138

